# Model-based machine learning

**DOI:** 10.1098/rsta.2012.0222

**Published:** 2013-02-13

**Authors:** Christopher M. Bishop

**Affiliations:** Microsoft Research, Cambridge CB3 0FB, UK

**Keywords:** Bayesian inference, graphical probabilistic programming, *Infer. NET*

## Abstract

Several decades of research in the field of machine learning have resulted in a multitude of different algorithms for solving a broad range of problems. To tackle a new application, a researcher typically tries to map their problem onto one of these existing methods, often influenced by their familiarity with specific algorithms and by the availability of corresponding software implementations. In this study, we describe an alternative methodology for applying machine learning, in which a bespoke solution is formulated for each new application. The solution is expressed through a compact modelling language, and the corresponding custom machine learning code is then generated automatically. This *model-based* approach offers several major advantages, including the opportunity to create highly tailored models for specific scenarios, as well as rapid prototyping and comparison of a range of alternative models. Furthermore, newcomers to the field of machine learning do not have to learn about the huge range of traditional methods, but instead can focus their attention on understanding a single modelling environment. In this study, we show how probabilistic graphical models, coupled with efficient inference algorithms, provide a very flexible foundation for model-based machine learning, and we outline a large-scale commercial application of this framework involving tens of millions of users. We also describe the concept of *probabilistic programming* as a powerful software environment for model-based machine learning, and we discuss a specific probabilistic programming language called *Infer.NET*, which has been widely used in practical applications.

## 1. Introduction

The origins of the field of machine learning go back at least to the middle of the last century. However, it was only in the early 1990s that the field began to have widespread practical impact. Over the last decade in particular, there has been a rapid increase in the number of successful applications, ranging from web search to autonomous vehicles, and from medical imaging to speech recognition. This has been driven by the increased availability of inexpensive computers, the development of improved machine learning algorithms, greater interest in the area from both the research community and the commercial sector, and most notably by the ‘data deluge’ characterized by an exponentially increasing quantity of data being gathered and stored on the world's computers.

During this time, large numbers of machine learning techniques have been developed, with names such as logistic regression, neural networks, decision trees, support vector machines, Kalman filters and many others. Contributions to this multi-disciplinary effort have come from the fields of statistics, artificial intelligence, optimization, signal processing, speech, vision and control theory, as well as from the machine learning community itself. In the traditional approach to solving a new machine learning problem, the practitioner must select a suitable algorithm or technique from the set with which they are familiar, and then either make use of existing software, or write their own implementation. If the technique requires modification to meet the particular requirements of their specific application, then they must be sufficiently familiar with the details of the software to make the required changes.

**Figure 1.**
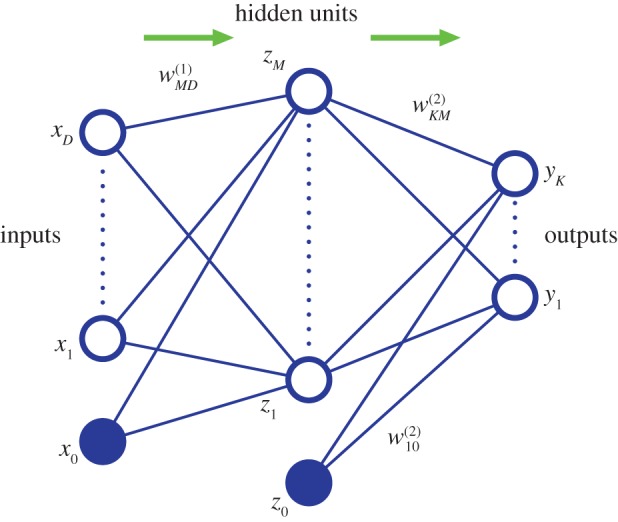
A neural network with two layers of adjustable parameters, in which each parameter corresponds to one of the links in the network. (Online version in colour.)

An example of a traditional machine learning technique is the two-layer neural network [1], illustrated diagrammatically in figure 1. The neural network can be viewed as a flexible nonlinear parametric function from a set of inputs {*x*_*i*_} to a set of outputs {*y*_*k*_}. First, linear combinations of the inputs are formed, and these are transformed using a nonlinear function *h*(⋅) so that
1.1
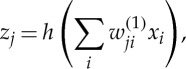
where *h*(⋅) is often chosen to be the ‘
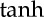
’ function. These intermediate variables are then linearly combined to produce the outputs
1.2
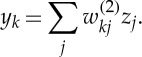
The variables 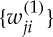 and 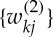 are the adjustable parameters of the network, and their values are set by minimizing an error function defined with respect to a set of training examples, each of which consists of a set of values for the input variables together with the corresponding desired values for the output variables. In a typical application of a neural network, the parameters are tuned using a training dataset, with the number of hidden units optimized using separate validation data. The network parameters are then fixed, and the neural network is then applied to new data in which the network makes predictions for the outputs given new values for the input variables.

A recent example of a very successful application of traditional machine learning is the skeletal tracking system in *Kinect* [2], which uses the signals from a depth video camera to perform real-time tracking of the full human skeleton on low-cost hardware. It is based on a technique known as *random forests of decision trees*, and the training data consists of one million depth images of human body poses, each of which is labelled with body parts (right hand, left knee, etc.). Again, this example follows a typical workflow for traditional machine learning applications. The parameters of the system, in this case, the selected features and thresholds at the nodes of the decision trees, as well as the depths of the trees themselves, are determined in the laboratory during the training phase. Once the performance of the system is satisfactory, the parameters are then fixed, and identical copies of the trained system are provided to its millions of users.

While the traditional approach to machine learning has resulted in numerous successful applications, and will undoubtedly continue to be an important paradigm for many years to come, it suffers from some notable limitations. Foremost of these is the difficulty of adapting a standard algorithm to match the particular requirements of a specific application. While some problems can be tackled using off-the-shelf machine learning methods, others will require appropriate modifications, which in turn requires an understanding both of the underlying algorithms and of the software implementation. Moreover, there are many applications for which it is difficult to cast a solution in the form of a standard machine learning algorithm. The Bayesian ranking problem, discussed in §6, in which the set of variables and their connectivity grows through time in a way that cannot be predicted in advance, is a good example.

Furthermore, the popularity and importance of machine learning means that it has moved beyond the domain of the machine learning community to the point where many researchers whose expertise lies in other fields, such as the physical and biological sciences, statistics, medicine, finance and many others, are interested in solving practical problems using machine learning techniques. The variety of algorithms, as well as the complex nomenclature, can make the field challenging for newcomers. More broadly, the ‘data revolution’ is creating many new opportunities for application developers to exploit the power of learning from data, few of whom will have a background in machine learning.

With the explosion in the quantity of data in the world, and the opportunities afforded by cloud computing whereby many datasets reside in data centres where they can be combined and where there is access to substantial computing resources, there is a significant opportunity to broaden the impact of machine learning. We therefore turn to an alternative paradigm for the creation of machine learning solutions, in order to address these issues. After summarizing the goals of model-based machine learning in §2, we show how these may be realized through the adoption of a Bayesian viewpoint (§3) coupled with probabilistic graphical models (§4) and deterministic approximate inference algorithms (§5). In §6, we consider a large-scale case study based on this framework, and in §7, we explain how *probabilistic programming* languages provide a powerful software environment for model-based machine learning, before drawing conclusions in §8.

## 2. Model-based machine learning

The central idea of the model-based approach to machine learning is to create a custom bespoke model tailored specifically to each new application. In some cases, the model (together with an associated inference algorithm) might correspond to a traditional machine learning technique, while in many cases it will not. Typically, model-based machine learning will be implemented using a model specification language in which the model can be defined using compact code, from which the software implementing that model can be generated automatically.

The key goals of a model-based approach include the following

— The ability to create a very broad range of models, along with suitable inference or learning algorithms, in which many traditional machine learning techniques appear as special cases.
— Each specific model can be tuned to the individual requirements of the particular application: for example, if the application requires a combination of clustering and classification in the context of time-series data, it is not necessary to mash together traditional algorithms for each of these elements (Gaussian mixtures, neural networks and hidden Markov models (HMMs), for instance), but instead a single, integrated model capturing the desired behaviour can be constructed.
— Segregation between the model and the inference algorithm: if changes are made to the model, the corresponding modified inference software is created automatically. Equally, advances in techniques for efficient inference are available to a broad range of models.
— Transparency of functionality: the model is described by compact code within a generic modelling language, and so the structure of the model is readily apparent. Such modelling code can easily be shared and extended within a community of model builders.
— Pedagogy: newcomers to the field of machine learning have only to learn a single modelling environment in order to be able to access a wide range of modelling solutions. Because many traditional methods will be subsumed as special cases of the model-based environment, there is no need for newcomers to study these individually, or indeed to learn the specific terminology associated with them.

A variety of different approaches could be envisaged for achieving the aims of model-based machine learning. In this study, we focus on a powerful framework based on Bayesian inference in probabilistic graphical models, and so we begin with a brief introduction to the Bayesian view of machine learning.

## 3. Bayesian inference

In many traditional machine learning methods, the adaptive parameters of the model are assigned point values that are determined by using an optimization algorithm to minimize a suitable cost function. By contrast, in a Bayesian setting, unknown variables are described using probability distributions, and the observation of data allows these distributions to be updated through Bayes' theorem. More generally, the Bayesian viewpoint involves the consistent quantification of uncertainty using probabilities. For each new observation or data point, the current distribution can be viewed as a *prior* distribution, from which Bayes' theorem allows the corresponding *posterior* distribution to be evaluated by incorporating the effect of the new data point. This posterior distribution in turn becomes the prior for use with the next observation. Note that this process is intrinsically sequential and is therefore well suited to online learning. Parameter optimization, which is widely used in traditional machine learning, is replaced in the Bayesian setting by *inference* in which the distributions over quantities of interest are evaluated, conditioned on the observed data.

A powerful feature of the Bayesian framework is the ease with which hierarchical models can be constructed. For example, we may wish to learn from data derived from a community of people while also personalizing the results for each individual by adapting to their specific data. This is readily achieved by using a model in which the individuals have their own parameter values whose prior distributions are governed by *hyper-parameters*, which themselves are drawn from a *hyper-prior* that is shared across the population.

Bayesian methods are at their most powerful when the supply of data is limited, and the resulting uncertainty in model parameters is significant. In such settings, traditional methods based on parameter optimization are prone to suffer from ‘over-fitting’, in which parameters are tuned to noise on the data, leading to poor predictions.

For large datasets, the probability distributions in a Bayesian model can, in some cases, become relatively narrow and the model can give results that are similar to those obtained using traditional point-based methods. Care must be taken, however, to understand the meaning of ‘large’ in this context. Here, the size of the dataset does not refer to its computational size, measured in bytes, but instead its statistical size in relation to the model being considered. For example, in a problem where it is necessary to predict the value of a single output variable *y* given the value of a single input value *x*, and where it is known that these two variables have a linear relationship with the addition of a low level of Gaussian noise, then a relatively modest number of data points (say 10–20) may be sufficient to give accurate predictions with little residual uncertainty because, in the absence of noise, just two points would be sufficient to determine the linear relationship. Such a dataset is computationally small but statistically large. By contrast, a dataset consisting of a million images, each of several mega-pixels, containing labelled objects (cars, bicycles, animals, etc.) will be computationally large. However, when used for object recognition, such a dataset may be statistically small in that it may contain only a tiny fraction of the possible combinations of object class, object size and orientation, object colour, lighting, occlusion and so on.

Many of the new applications for machine learning arising from the data explosion are characterized by datasets that are computationally large but statistically small. There is therefore a need to develop methods for Bayesian inference that are computationally efficient and that scale well to computationally large datasets. Before discussing such methods, we first introduce a graphical framework that can be used to construct models.

## 4. Probabilistic graphical models

In a Bayesian setting, a ‘model’ consists of a specification of the joint distribution over all of the random variables in the problem
4.1
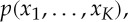
where {*x*_1_,…,*x*_*K*_} includes any ‘parameters’ in the model as well as any latent (i.e. hidden) variables, along with the variables whose values are to be observed or predicted. Working with fully flexible joint distributions is, in general, intractable, and inevitably we must deal with structured models. One very flexible framework for specifying such structure is given by *probabilistic graphical models* [3,4]. In this study, we focus on a particular form of graphical model based on *directed acyclic graphs*. These represent a pictorial way of expressing how the joint distribution is factored into the product of distributions over smaller subsets of variables.

**Figure 2.**
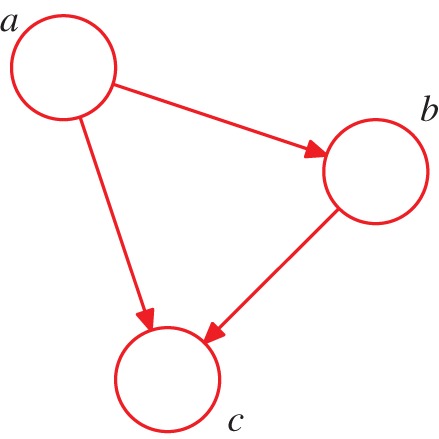
A directed graphical model representing the joint probability distribution over three variables *a*, *b* and *c*, corresponding to the decomposition on the right-hand side of (4.2). (Online version in colour.)

Consider a general distribution over three variables *a*, *b* and *c*. Using the product rule of probability [1], this can be factorized, without loss of generality, in the form
4.2
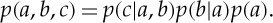
Here, the notation *p*(*x*|*y*) denotes a conditional probability in which the distribution of *x* depends on the value of *y*. Note that we have not yet specified whether these variables are continuous or discrete, nor have we specified the functional form of the various factors on the right-hand side of (4.2), such as Gaussian, Bernoulli or gamma distributions. The decomposition is therefore very general and applies to a whole family of models. We now represent the right-hand side of (4.2) in terms of a simple graphical model as shown in figure 2. To construct this graph, we first introduce a node for each of the random variables *a*, *b* and *c*, and associate each node with the corresponding conditional distribution on the right-hand side of (4.2). Then, for each conditional distribution, we add directed links (arrows) from whichever other nodes correspond to the variables on which that distribution is conditioned. Thus, for the factor *p*(*c*|*a*,*b*), there will be links from nodes *a* and *b* to node *c*, for the factor *p*(*b*|*a*), there is a single link from node *a* to node *b*, and for the factor *p*(*a*), there will be no incoming links. If there is a link going from a node *a* to a node *b*, then we say that node *a* is the *parent* of node *b*, and we say that node *b* is the *child* of node *a*.

**Figure 3.**
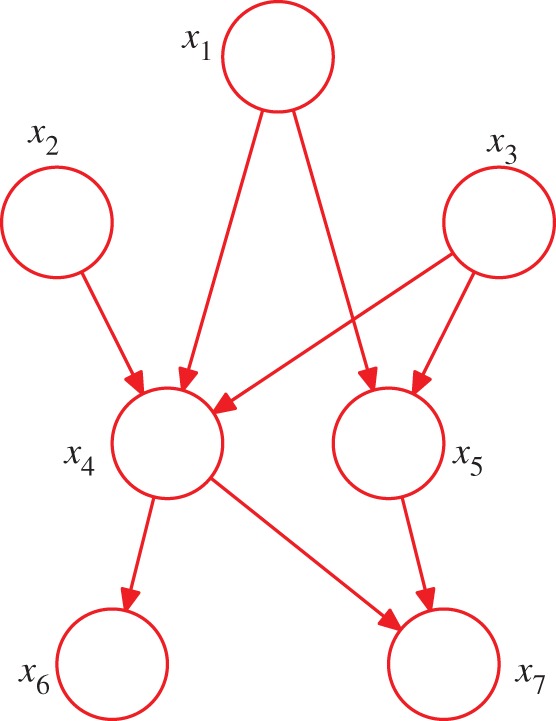
A directed acyclic graph over seven variables. This graph expresses a decomposition of the joint distribution given by (4.3). (Online version in colour.)

So far, we have worked with completely general joint distributions, so that the decompositions, and their representations as fully connected graphs, will be applicable to any choice of distribution. However, it is the *absence* of links in the graph that conveys interesting information about the properties of the class of distributions that the graph represents. Consider the graph shown in figure 3. This graph represents a factorization of the joint probability distribution in terms of the product of a set of conditional distributions, one for each node in the graph. Each such conditional distribution will be conditioned only on the parents of the corresponding node in the graph. For instance, *x*_5_ will be conditioned on *x*_1_ and *x*_3_. The joint distribution of all seven variables is therefore given by
4.3
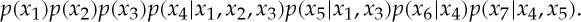
This is not a fully connected graph because, for instance, there is no link from *x*_1_ to *x*_2_ or from *x*_3_ to *x*_7_.

This factorization is readily extended to *K* variables, in which the joint distribution is given by
4.4
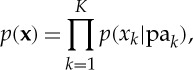
where pa_*k*_ denotes the set of parents of *x*_*k*_, and ***x***={*x*_1_,…,*x*_*K*_}.

The key point of this representation is that it allows the joint distribution over the potentially very large set of variables in the problem (millions of variables in some applications) to be expressed in terms of the product of factors, each of which typically depends only on a small subset of variables. This produces a substantial computational simplification and renders the models tractable. Analogous simplifications are a key aspect of traditional machine learning methods as well.

In the approach to model-based machine learning discussed in this study, we construct a probabilistic model expressed as a directed graph. The structure of the graph captures our assumptions about the plausible class of distributions that could be relevant to our application. The easiest way to understand the interpretation of the graph is to imagine generating synthetic data by *ancestral sampling* from the graph. This is called the *generative* viewpoint, and can be illustrated by considering figure 3. We draw a sample at each of the nodes in order, using the probability distribution at that node. This starts by drawing a value from the distribution *p*(*x*_1_), so that the random variable *x*_1_ takes a specific value 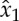. Likewise for 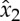 and 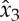. Next, *x*_4_ is sampled from 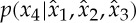, in which the parent variables are set to their sampled values. This process is continued until we have a sampled value for each of the variables.

**Figure 4.**
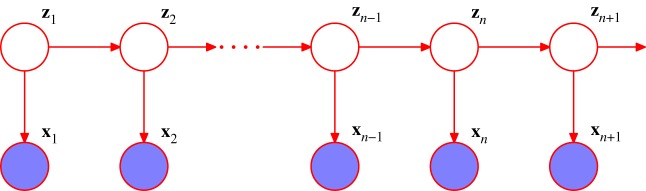
Graphical model representation of a hidden Markov model. This same graph also represents a linear dynamical system. Here, the shaded nodes represent *observed* variables, i.e. ones whose values are fixed by the dataset. (Online version in colour.)

As a specific example of a graphical model, consider the HMM [5], which can be represented using the probabilistic graphical model shown in figure 4. This model is widely used in speech recognition [6], natural language modelling [7], analysis of biological sequences [8] and many other fields. The HMM can be applied to datasets that consist of a sequence of observed vectors ***x***_1_,***x***_2_,…. The model assumes that there is a latent (hidden) process involving a Markov chain of unobserved discrete variables ***z***_1_,***z***_2_,…. Each observed value *x*_*k*_ depends only on the latent variable ***z***_*k*_ at the same time step. Inference in this model can be done efficiently using the *forward–backward* algorithm [5]. It is also possible to consider the same graphical structure but with continuous latent variables based on Gaussian distributions. In the case of figure 4, this leads to *linear dynamical systems* [9]. Inference for this model corresponds to the Kalman filter and the Kalman smoother algorithms [1,10,11].

One of the most powerful aspects of probabilistic graphical models is the relative ease with which a model can be customized to a specific application, or modified if the requirements of the application change. This can be illustrated by looking at some variants of the HMM.

**Figure 5.**
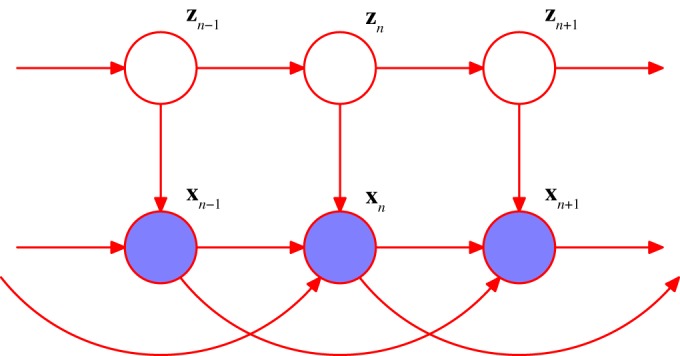
An extension of the model in figure 4 to include auto-regressive dependencies. (Online version in colour.)

**Figure 6.**
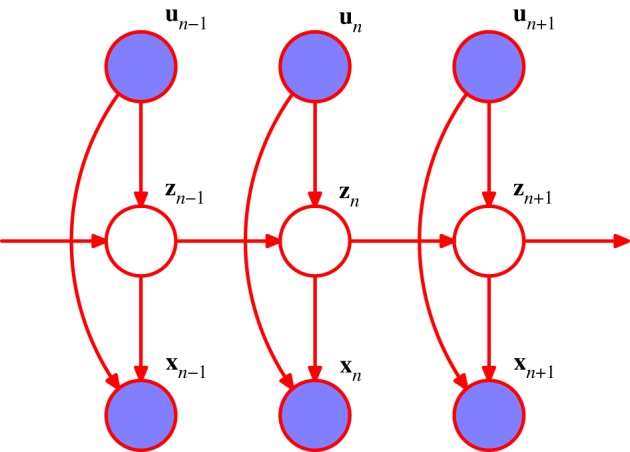
An extension of the model in figure 4 to include input variables as well as outputs. (Online version in colour.)

**Figure 7.**
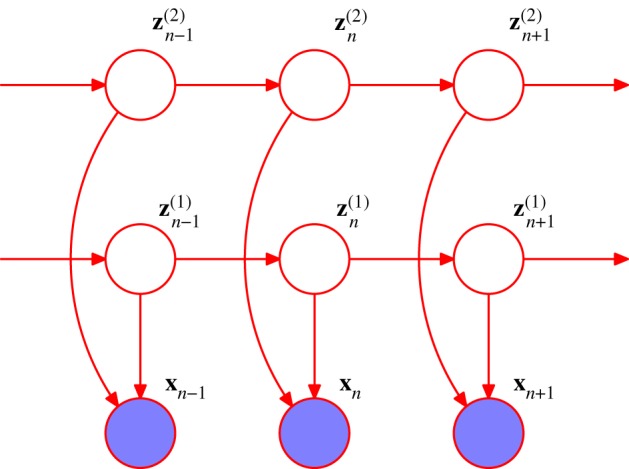
An extension of the model in figure 4 for multiple hidden Markov processes. (Online version in colour.)

One possible extension to the basic HMM involves the inclusion of additional links to give an *autoregressive HMM*, as shown in figure 5. In this model, the observed value ***x***_*n*_ at step *n* depends not only on the hidden variable ***z***_*n*_, but also on previous observed values. Another development of the HMM is to include ‘inputs’ as well as ‘outputs’, for example, using the graphical structure shown in figure 6. Yet another variant is the *factorial HMM* [12], shown in figure 7. Here, there are multiple hidden processes (only two are shown in the case of figure 7), and the output at a particular time step depends on all of the hidden states at that time. This can be viewed as a special case of an HMM with restricted structure in the hidden process, and this structure can be exploited to give more efficient inference. An interesting development of this idea is the *switching state-space model* [13], in which there are multiple independent Markov chains of latent variables, and the distribution of the observed variable at a given time step is conditional on the state of only one of those chains. The particular chain responsible at each step is itself determined by the state of another discrete hidden Markov process. The key point here is that many variants are possible, and in particular a new model can readily be developed that is tailored to each particular application.

A high proportion of the standard techniques used in traditional machine learning can be expressed as special cases of the graphical model framework, coupled with appropriate inference algorithms. For example, principal component analysis (PCA), factor analysis, logistic regression, Gaussian mixtures and similar models can all be represented using simple graphical structures. These can then readily be combined, for example, to form a mixture of probabilistic PCA models. To construct and use these models within a model-based machine learning framework, it is not necessary to know their names or be familiar with the specific literature on their properties.

Note that for the detailed design of models, it is often more convenient to use a richer graphical framework called *factor graphs* [1,14], which can represent a superset of directed graphs. Owing to lack of space, we will not discuss factor graphs further in this article.

So far we have assumed that the structure of the graph is determined by the user. In practice, there may be some uncertainty over the graph structure, for example, whether particular links should be present or not, and so there is interest in being able to determine such structure from data. A powerful graphical technique to help with this is called *gates* [15], which allows random variables to switch between alternative graph structures, thereby introducing a higher-level graph that implicitly includes multiple underlying graph structures. Running inference on the gated graph then gives posterior distributions over different structures, conditioned on the observed data.

## 5. Approximate inference algorithms

As we have seen, a probabilistic model defines a joint distribution over all of the variables in our application. We can partition these variables into those that are observed ***x*** (the data), those whose value we wish to know ***z***, and the remaining latent variables *w*. The joint distribution can therefore be written as *p*(***x***,***z***,***w***). If we had not observed ***x***, then the *marginal* distribution over ***z*** would be given by
5.1
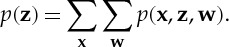
Here, we assume that the variables are discrete, but the discussion in this study applies equally to continuous variables, or to a combination of discrete and continuous variables, in which case, the summations are replaced, where appropriate, by integrations.

Observing that ***x*** takes a specific value 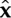 allows us to compute the conditional distribution
5.2
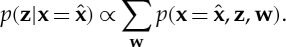
Here, the notation 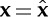 denotes that the random variable ***x*** takes the specific value 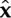. If desired, the resulting distribution can be normalized. We can view (5.1) as a prior distribution defined before the data are observed, with (5.2) as the corresponding posterior distribution. The change in distribution in going from the prior to the posterior reflects the information gained as a result of observing the data, and represents the modern Bayesian perspective on what it means for a machine to ‘learn from data’.

In most applications, we limit our attention to the determination of the posterior marginals of individual variables of the form
5.3
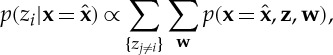
for each of the variables *z*_*i*_ that comprise ***z***.

For essentially all problems of practical interest, the exact evaluation of (5.2) or (5.3) is infeasible. We must therefore resort to approximations, which themselves need to be computationally efficient while achieving sufficient accuracy for the particular application.

Let us begin by looking at the question of computational efficiency. Consider the case of a model with *M* discrete latent variables comprising the vector ***w***, each having cardinality *K*. The summation over ***w*** in (5.2) then involves *K*^*M*^ terms, and so the storage and computational requirements grow exponentially with the number of variables. Even for binary variables, this becomes intractable for many real-world applications, which may involve thousands or millions of variables.

We can often improve the situation dramatically by making use of structure within the model. Consider a model specified by a directed graph, in which the joint distribution has a factorization specified by (4.4). If the individual factors depend only on small subsets of the variables, then we can exploit the factorization to obtain a more efficient inference procedure. To illustrate this, consider a toy example involving two binary variables *a* and *b*, and a function given simply by the product *ab*. If we sum this function over all values of *a* and *b*, we obtain
5.4
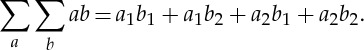
We see that evaluation of the right-hand side requires seven operations (four multiplications and three additions). However, we can exploit the fact that the function *ab* factorizes into the product of a function of *a* and a function of *b* to enable us to rewrite (5.4) in the analytically equivalent form
5.5
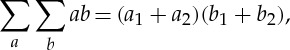
which now only requires three computational steps (two additions and one multiplication). We have exploited the factorization structure to exchange summation and multiplication and thereby achieve a form that is analytically equivalent but computationally more efficient.

**Figure 8.**
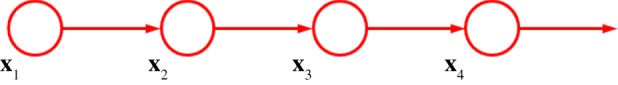
A simple Markov chain of variables. (Online version in colour.)

Now consider a more complex example consisting of a chain of nodes, as shown in figure 8. Again, suppose the chain has *M* discrete variables each of cardinality *K*, and that we wish to calculate the marginal distribution of ***x***_*M*_. A naive calculation would involve evaluation of the joint distribution and then marginalization over the unwanted variables
5.6
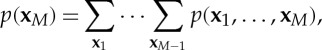
which, if evaluated directly, incurs storage and computational costs that are both exponential in the length of the chain.

To obtain a more efficient inference procedure, we make use of the factorization of the joint distribution, given by
5.7
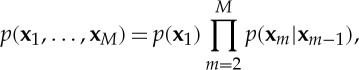
which is obtained by applying (4.4) to the graph in figure 8. By substituting (5.7) into (5.6), and exchanging the order of summations and products, we obtain
5.8
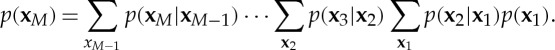
Here, the sum over ***x***_1_ is evaluated first, and involves only the distributions *p*(***x***_2_|***x***_1_) and *p*(***x***_1_). This step therefore requires storage and computation that is only *O*(*K*^2^). The resulting quantity is a function only of ***x***_2_ and is then multiplied by *p*(***x***_3_|***x***_2_) and then summed over ***x***_2_, which again is *O*(*K*^2^) in computation and storage. The process is repeated down the chain, giving an overall computational cost that is *O*(*MK*^2^). Thus, by using the factorization of the joint distribution, we have reduced the computation from one that is exponential in the length of the chain to one that is linear in the length of the chain. Note that this is still an exact calculation.

This procedure can be interpreted as a *message-passing scheme* in which the quantity
5.9
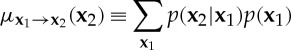
can be viewed as a message being sent from node ***x***_1_ in the graph to node ***x***_2_. Similarly, a general step in the calculation can be expressed as the evaluation of an outgoing message that is constructed from an incoming message combined with a local conditional distribution
5.10
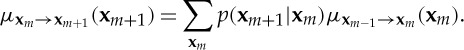
Thus, the global calculation can be broken down into local calculations involving messages passed between adjacent nodes in the chain. In this particular example, a sequence of messages is passed from one end of the chain to the other.

This approach can readily be generalized to an arbitrary graph that has no loops [3,1]. In this case, the marginal distributions of all of the unobserved nodes can be evaluated using a two-stage message-passing schedule as follows. Any one of the nodes is first designated as the ‘root’. Messages are then passed sequentially out from the root via all other nodes to the ‘leaves’. A second set of messages is then passed from the leaf nodes back to the root node. At the end of this second pass, each link will have seen one message pass in each direction, and each node will have received sufficient information to be able to compute its marginal distribution, conditioned on any observed variables. Again, the computational cost scales linearly in the size of the graph. A particular instance of this algorithm is the *forward–backward algorithm* for finding the posterior marginals in an HMM, used to learn the parameters of the model [5]. Another special case is given by the Kalman filter (forward pass) and Kalman smoother (backward pass) algorithms for linear dynamical systems [11,1].

For graphs with loops, the situation is more complex. Exact inference can still be performed using techniques such as the *junction tree algorithm* [16], but the computational cost can become prohibitive, depending on the structure of the graph. An alternative approach, known as *loopy belief propagation* [17], uses the same message-passing technique as discussed earlier for tree-structured graphs, but simply iterates the messages to allow for the fact that, with loops present, a standard two-pass schedule does not lead to exact marginals. Although this process may seem ad hoc, it has been found to yield good results in many applications.

So far, we have assumed that the local messages at each node can be computed exactly. While this is typically true for discrete nodes, for other distributions, a closed-form evaluation of the messages is often not possible, and it becomes necessary to resort to approximations. One class of approximation scheme is based on sampling using techniques such as *Markov chain Monte Carlo* (MCMC) [18]. A very simple, though widely applicable, MCMC method is *Gibbs sampling*. Two advantages of Monte Carlo methods are their broad applicability to a wide range of distributions, and that many of them asymptotically give exact inference in the limit of infinite compute resources. In practice, however, Monte Carlo methods are computationally expensive, and typically do not scale to the large datasets encountered in many technological applications, particularly those involving internet-scale datasets. We therefore turn instead to an alternative class of inference algorithms based on deterministic approximations.

Here, we consider a specific approximation framework called *expectation propagation* [19]. The local messages are approximated through minimization of the Kullback–Leibler (KL) divergence given by
5.11
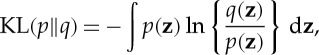
where *q*(***z***) represents a family of approximating distributions. The KL divergence measures the extent to which the distribution *q*(***z***) differs from the given distribution *p*(***z***), and has the property *KL*(*p*∥*q*)≥0, with equality if, and only if, *q*(⋅)=*p*(⋅). We shall see an example of the application of this procedure in the next section. For graphs with loops, the message-passing procedure can again be continued iteratively until some stopping criterion is satisfied.

There are many other deterministic approximation schemes such as *variational message passing* [20], *tree-reweighted message passing* [21], *fractional belief propagation* [22] and *power EP* [23]. Furthermore, it has been shown [24] that a broad range of message-passing algorithms can be derived from a common framework involving the minimization of a generalization of the KL divergence known as the *alpha family* of divergences.

## 6. Case study: Bayesian skill rating

We now consider a real-world example of the application of the framework of graphical models and approximate deterministic inference discussed in the previous sections. The model is known as *TrueSkill* [25], and it addresses the problem of determining the skill ratings of players in a series of competitive games. It generalizes the widely used Elo system [26] that is used, for example, in international chess gradings. TrueSkill was deployed on the *Xbox Live* online gaming system in 2005, and has been operating continuously since then, processing millions of game outcomes per day.

The goal is to assign a skill value to each of the players on the basis of game outcomes. Because the skill *s*_*i*_ of player *i* is unknown, in the Bayesian setting, it is assigned a probability distribution that, for simplicity, is given by a Gaussian with mean *μ*_*i*_ and variance 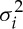. Under the Elo system, it is usual to regard a player's rating as provisional until a sufficient number of games (say 20) has been played. This issue does not arise in a Bayesian setting since the uncertainty in the player's skill is quantified from the start. As new data (i.e. new game results) arrive, the skill distribution is updated, and a reduction in the variance of this distribution represents increasing confidence in the value of the player's skill.

**Figure 9.**
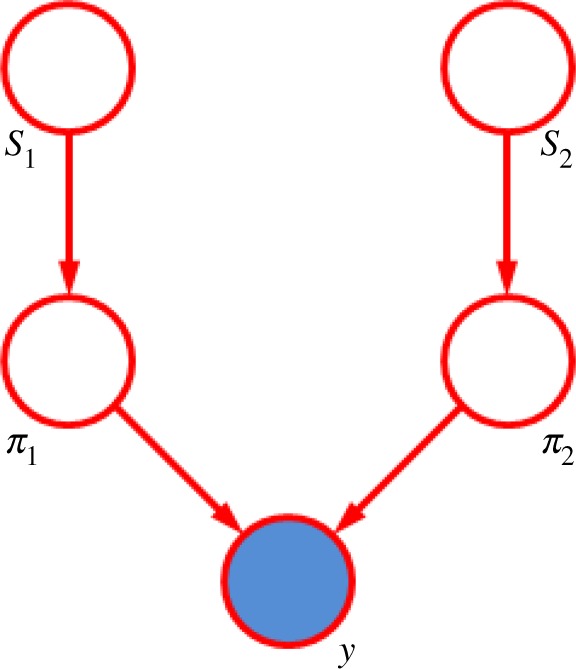
Directed graph showing the TrueSkill model for a single game between two players. See the text for details. (Online version in colour.)

Consider a specific game between player 1 and player 2. We define for each player a *performance* *π*_*i*_ that represents how well they played on that particular game. Because the performance of a player with a given skill can vary from game to game, the performance is a noisy version of the skill. This is represented by giving *π*_*i*_ a Gaussian distribution, whose mean is *s*_*i*_ and with a variance *β*. The winner of the game is the player with the higher performance value. This can be represented by introducing a variable *y*=*π*_2_−*π*_1_, where *y*>0 indicates that player 2 is the winner. Draws can also be modelled as occurring when the difference in performance values is below a threshold |*y*|≤*ϵ*. The overall graphical model for this specific game is shown in figure 9.

When the game outcome is known, the node *y* becomes observed, and the inference problem involves updating the distributions over the skills *s*_1_ and *s*_2_. For this model, the graph is tree structured. However, the exact messages from the node *y* are non-Gaussian, and so the posterior distribution over skills becomes non-Gaussian. The messages are therefore approximated using expectation propagation, in which the exact distribution is replaced by the Gaussian distribution that locally minimizes the KL divergence (5.11). This ensures that the distributions remain within the exponential family. The required distribution can be calculated using moment-matching, i.e. by matching the mean and variance of the approximating Gaussian to the corresponding values for the true distribution. Note that this Bayesian model is intrinsically sequential, with the posterior skill distributions acting as the priors for the next round of inference once new data are observed. The current skill distributions are used to select opponents in the online gaming environment, and the results of the corresponding games are then used to make further refinement of the skill distributions. Thus, inference and decision are interleaved, and the graphical model is being continuously created. This is a far cry from the traditional machine learning paradigm in which the parameters of a model are tuned in the laboratory using a training dataset (with *cross-validation* to avoid over-fitting), the parameters then frozen and the fixed system used to make predictions on future test data.

**Figure 10.**
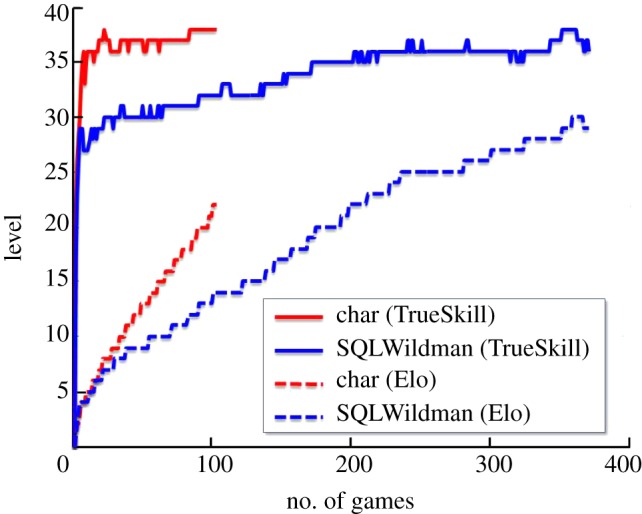
Graph of skill levels for two players in an online game, showing the much faster convergence obtained using TrueSkill compared to the traditional Elo algorithm. (Online version in colour.)

Figure 10 shows some results obtained with TrueSkill, along with the corresponding results using Elo. Here, we see the estimated skill level from Elo for two players in an online computer game plotted against the number of games played. Also plotted are the posterior mean skills for the two players obtained from TrueSkill using the same data, showing the much faster convergence as a function of the number of games played. This improved behaviour is a consequence of the Bayesian modelling of uncertainty in TrueSkill, in which each player has a mean and variance for their skill level, compared with the single estimated skill value in Elo [25].

**Figure 11.**
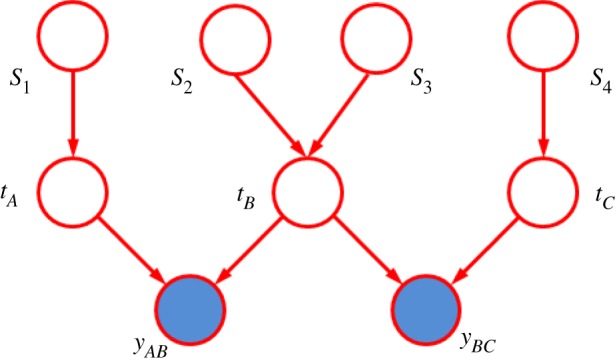
Modified skill rating graph showing the inclusion of three teams *A*, *B* and *C*, in which team *B* has two players. (Online version in colour.)

One of the powerful aspects of model-based machine learning is the ability to extend the model to take account of more complex situations. To illustrate this, consider two of the significant limitations of the conventional Elo system: (i) game outcomes often refer to teams of players, yet for matchmaking purposes we need the skills of individual players, and (ii) many games involve more than two players (or more than two teams of players). These limitations are significant in the context of online computer games, and can be overcome in TrueSkill by a simple extension of the model, as shown by the graph in figure 11. Note that, with more than two players, the message-passing algorithm must be run iteratively until a suitable stopping criterion is met.

Further extensions to the model are easily made. For example, we can take account of the evolution of a player's skill through time (e.g. as a result of gaining experience) by introducing some Gaussian diffusion in the spirit of the Kalman filter. Again, this is easily accommodated by modifying the underlying probabilistic graph [25,27].

## 7. Probabilistic programming

In this study, we have outlined a framework for model-based machine learning built on approximate Bayesian inference in graphical models using local message-passing algorithms. In order to apply this framework in practice, we need appropriate software development tools. A very flexible environment for model-based machine learning is known as *probabilistic programming* [28]. This can be viewed as an extension of classical programming to include random variables as first-class citizens alongside conventional deterministic variables, in which standard operators are overloaded, allowing them to manipulate both deterministic and random variables. The random variables themselves might be represented in terms of specific distributions, for example, from the exponential family, or using some non-parametric or sample-based representation.

**Figure 12.**
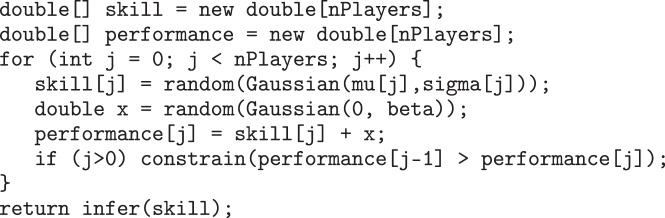
Csoft code for the TrueSkill model.

We can illustrate the key ideas of probabilistic programming using *Csoft* (J. Winn & T. Minka 2012, personal communication), which is an extension of the C# programming language to include support for random variables. Three new features are required. First, random variables can be defined using the keyword ‘random’, for example,

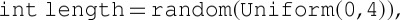
which says that length is a random variable that is uniformly distributed over the interval (0,4). Second, constraints involving random variables can be including using the ‘constrain’ keyword, as in

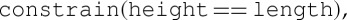
which says that the random variable height must be equal to the random variable length. Similarly, we can constrain random variables to take on specific values, as in

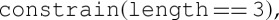
which would be used to set random variables to their observed values and hence to incorporate data into a model. Finally, the distributions of random variables can be obtained using the ‘infer’ keyword, for example,

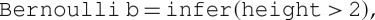
which returns a Bernoulli distribution giving the probability that the random variable height takes a value greater than 2. Figure 12 shows the Csoft code corresponding to the TrueSkill model discussed in §6.

A language such as Csoft allows probabilistic and conventional deterministic code to be combined, and provides a flexibility of modelling that goes beyond conventional graphical model notation. For example, jagged arrays can capture a complex sparse connectivity structure that is difficult to express succinctly in the standard graphical formalism.

Conceptually, we can interpret a probabilistic program from a sampling perspective. For each occurrence of random, we draw a sample from the corresponding distribution; for each occurrence of constrain, the program terminates if the constraint is violated; and for each occurrence of infer, the program collects the values of the required variables into a persistent memory. If the code is then run a large number of times, the persistent memory accumulates a sample-based representation of the required distributions. Obviously, this ‘rejection sampling’ technique is too slow for most practical applications, and more efficient inference techniques are required, for example, based on local message passing.

**Figure 13.**
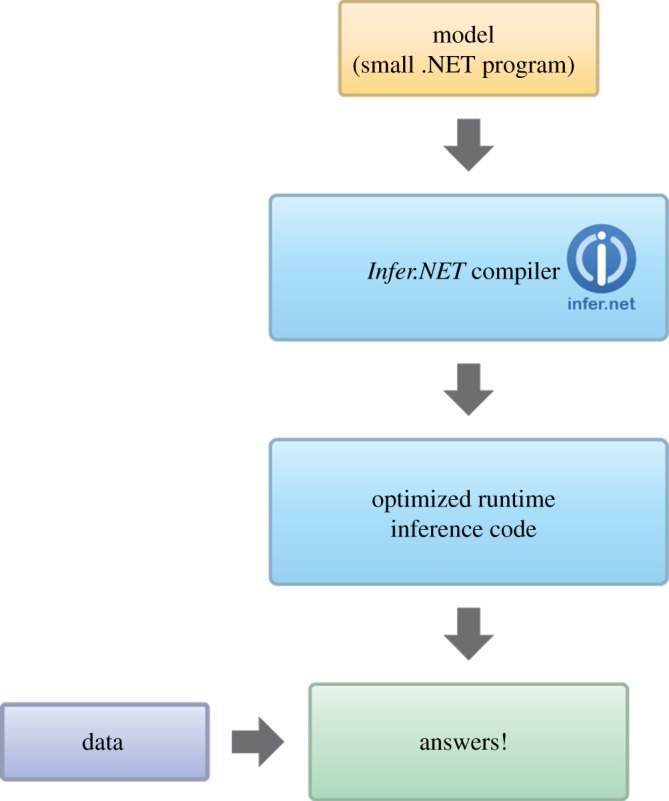
Flow diagram showing the operation of *Infer.NET*. (Online version in colour.)

An example of a probabilistic programming language is *Infer.NET* [29]. This supports a wide range of distributions involving both discrete and continuous variables, and has a modular framework that is readily extended to new distributions. Typically, we expect general-purpose software to have a computational efficiency that is poor compared with model-specific software. However, *Infer.NET* is able to achieve efficiency that is often close to hand-tuned code, by adopting a compiler technology as illustrated in figure 13. Note that in this diagram, the .NET program that specifies the ‘model’ includes a description of which variables are observed (but without the values of those observations). This allows the compiler to generate inference code that is optimized for the particular partition of observed and hidden variables. In some applications, it might not be known which variables will be observed until run time, and in such cases, the model can be extended with additional variables that allow for observing the partition at run time. For example, a model could be extended to include binary variables specifying, for each potentially observable variable, whether or not that variable is in fact observed. The *Infer.NET* compiler encapsulates numerous optimizations regarding the choice of message-passing schedule in order to generate efficient inference code. Currently, *Infer.NET* supports two deterministic inference algorithms (expectation propagation and variational message passing), as well as a Monte Carlo method (Gibbs sampling).

Another probabilistic programming language, with some similarities to *Infer.NET*, is Bayesian inference using Gibbs sampling (*BUGS*) [30]. *BUGS* uses Monte Carlo methods, which give it great flexibility in the range of models that it can accommodate, but owing to the computational cost of Monte Carlo inference, it does not scale well to large datasets. There are many other languages currently in development, and probabilistic programming has become a very active field of research.

## 8. Conclusions

In this study, we have given an overview of the model-based approach to machine learning, and discussed its advantages compared with traditional approaches, including the ability to develop custom models that are optimized for each application. We have outlined a particular framework for model-based machine learning based on deterministic inference in probabilistic graphical models using local message-passing algorithms. We have also discussed a very general software development environment for model-based machine learning called probabilistic programming, and described a specific instantiation in the form of *Infer.NET*. Model-based machine learning, particularly in the form of probabilistic programming, is a highly active field of research, and offers great potential to capitalize on the new era of data-driven computing.
